# Meta-analysis of HIV-1 vaccine elicited mucosal antibodies in humans

**DOI:** 10.1038/s41541-021-00305-8

**Published:** 2021-04-15

**Authors:** Kelly E. Seaton, Aaron Deal, Xue Han, Shuying S. Li, Ashley Clayton, Jack Heptinstall, Ann Duerr, Mary A. Allen, Xiaoying Shen, Sheetal Sawant, Nicole L. Yates, Paul Spearman, Gavin Churchyard, Paul A. Goepfert, Janine Maenza, Glenda Gray, Giuseppe Pantaleo, Laura Polakowski, Harriet L. Robinson, Shannon Grant, April K. Randhawa, Ying Huang, Cecilia Morgan, Nicole Grunenberg, Shelly Karuna, Peter B. Gilbert, M. Juliana McElrath, Yunda Huang, Georgia D. Tomaras, NIAID HIV Vaccine Trials Network (HVTN) 076, 088, 086, 096, 097, 205 Study Teams

**Affiliations:** 1 Duke Human Vaccine Institute, Durham, NC USA; 2 Department of Surgery, Duke University, Durham, NC USA; 3 Department of Immunology, Duke University, Durham, NC USA; 4 Department of Molecular Genetics and Microbiology, Duke University, Durham, NC USA; 5 Vaccine and Infectious Disease Division, Fred Hutchinson Cancer Research Center, Seattle, WA USA; 6 Division of AIDS, NIAID, NIH, Bethesda, MD USA; 7 Division of Infectious Diseases, Cincinnati Children’s Hospital, Cincinnatti, OH USA; 8 Aurum Institute, Johannesburg, South Africa; 9 School of Public Health, University of Witwatersrand, Johannesburg, South Africa; 10 Department of Medicine, University of Alabama at Birmingham, Birmingham, AL USA; 11 Division of Allergy and Infectious Diseases, Department of Medicine, University of Washington, Seattle, WA USA; 12 South African Medical Research Council, Cape Town, South Africa; 13 Service of Immunology and Allergy, and Swiss Vaccine Research Institute, Lausanne University Hospital, Lausanne, Switzerland; 14 GeoVax Labs, Inc., Smyrna, GA USA; 15 Department of Biostatistics, University of Washington, Seattle, WA USA; 16 Department of Global Health, University of Washington, Seattle, WA USA

**Keywords:** DNA vaccines, Protein vaccines, Vaccines, Immunology, Infectious diseases

## Abstract

We studied mucosal immune responses in six HIV-1 vaccine trials investigating different envelope (Env)-containing immunogens. Regimens were classified into four categories: DNA/vector, DNA/vector plus protein, protein alone, and vector alone. We measured HIV-1-specific IgG and IgA in secretions from cervical (*n* = 111) and rectal swabs (*n* = 154), saliva (*n* = 141), and seminal plasma (*n* = 124) and compared to corresponding blood levels. Protein-containing regimens had up to 100% response rates and the highest Env-specific IgG response rates. DNA/vector groups elicited mucosal Env-specific IgG response rates of up to 67% that varied across specimen types. Little to no mucosal IgA responses were observed. Overall, gp41- and gp140-specific antibodies dominated gp120 mucosal responses. In one trial, prior vaccination with a protein-containing immunogen maintained durability of cervical and rectal IgG for up to 17 years. Mucosal IgG responses were boosted after revaccination. These findings highlight a role for protein immunization in eliciting HIV-1-specific mucosal antibodies and the ability of HIV-1 vaccines to elicit durable HIV-1-specific mucosal IgG.

## Introduction

The majority of HIV-1 infections occur through mucosal routes such that inducing protective antibodies within the mucosa is an important goal for preventive HIV-1 vaccine development. Therefore, investigating the nature of HIV-1-specific antibody responses at mucosal sites post-vaccination is key to designing vaccines that elicit protective immunity. HIV-1 infection elicits mucosal antibodies with functional antiviral activity, including broadly neutralizing antibodies (bnAb) and non-bnAb specificities^1,2^, demonstrating the capacity of the immune system to generate and traffic functional anti-HIV antibodies to mucosal surfaces. Studies conducted in nonhuman primates (NHP) demonstrate protection against mucosal simian-human immunodeficiency virus (SHIV) challenge by applying bnAbs to mucosal surfaces^3^ or through the systemic, passive infusion of bnAbs that penetrate the mucosal surfaces^4–11^. bnAb-mediated protection is thought to also involve antibody Fc effector functions and may help clear infected cells after mucosal challenge^12^. Furthermore, passively infused non-neutralizing antibodies with antibody effector functions can impact virus transmission at the mucosa^13–15^. Antibody combinations (e.g., IgA and IgG) present at the mucosa may act together to prevent transmission^16^. In a recent preclinical NHP model of vaccine-elicited protection against SHIV acquisition utilizing a combination of a viral vector and protein immunogen with potent adjuvants, the concentration of vaginal gp140 antibodies on the day of challenge correlated with the number of challenges needed for infection^17^. Moreover, vaccine induction of homologous tier 2 neutralizing antibodies^18^ protected against intra-rectal SHIV challenge. These encouraging results for protective vaccine-elicited antibody responses in NHP provide the rationale for examining levels of vaccine-induced mucosal antibodies in human studies and optimizing regimens to improve mucosal antibody responses.

We performed an individual-level meta-analysis of multiple vaccine regimens and multiple sample types across systemic and mucosal compartments. Serum and mucosal specimens, including cervical and rectal secretions, seminal plasma, and saliva, were analyzed using a binding antibody multiplex assay to determine the magnitude, specificity, and durability of vaccine-induced, HIV-specific antibodies. Results from this study answer several key questions regarding vaccine-elicited mucosal antibodies, including the elicited isotypes and the effect of vaccine type (DNA/vector, DNA/vector + protein, protein only, and vector-only) on antibody response rate and magnitude. We also investigated whether gp41 antibodies are preferentially elicited in vaccine regimens containing a gp140 component and whether mucosal antibody responses are correlated with serum responses and across mucosal compartments. These results will inform vaccine design aimed at eliciting antiviral antibodies at primary sites of HIV-1 exposure.

## Results

### Meta-analysis of human HIV-1 vaccine regimens includes DNA and vector prime with protein boost immunogens

We conducted a meta-analysis of vaccine-elicited mucosal antibodies in six clinical trials of HIV-1 vaccines containing envelope in the prime and/or boost immunogens (Table 1). These included HVTN 076 (VRC DNA/Ad5), HVTN 205 (pGA2/JS7 DNA and/or MVA/HIV62)^19^, HVTN 088 (Clade C gp140/MF59 delayed boosts following previous receipt of DNA or vector + protein/MF59 HIV vaccines [“primed”] or in HIV vaccine naïve persons [“unprimed”])^20^, HVTN 086 (SAAVI DNA-C, MVA-C, Novartis clade C gp140/MF59)^21^, HVTN 096 (NYVAC-C/DNA-C/AIDSVAX B/E)^22^ and HVTN 097 (ALVAC/AIDSVAX B/E)^23,24^. We classified the vaccine regimens from these trials into four broad vaccine types: DNA/vector, DNA/vector + protein, protein only, and vector only, and pooled placebos from all trials into one placebo group. Mucosal sampling included secretions from cervical (*n* = 111) and rectal swabs (*n* = 154), saliva (*n* = 141), and seminal plasma (*n* = 124) (Table 1).

**Table 1:** HIV-1 vaccine regimen and dosing schedules.

	Protocol	Arm	VAC1	VAC2	VAC3	VAC4	Env	Specimens Analyzed
DNA/vector	HVTN 076 NCT00955006	T1	DNA	DNA	DNA	Ad5	gp140	CS (*n* = 6) Saliva (*n* = 16) SP (*n* = 10)
	HVTN 086 NCT01418235	T3	DNA + placebo	DNA	MVA + placebo	MVA + placebo	gp150	CS (*n* = 4) RS (*n* = 7)
	HVTN 205 NCT00820846	T1 + T3	DNA	DNA	MVA	MVA	gp160, gp150	CS (*n* = 6) RS (*n* = 7) SP (*n* = 9)
DNA/vector + protein	HVTN 086 NCT01418235	T1	MVA + placebo	MVA	gp140/MF59 + placebo	gp140/MF59 + placebo	gp150, gp140	CS (*n* = 9) RS (*n* = 9)
		T4	DNA + placebo	DNA	MVA + gp140/MF59	MVA + gp140/MF59	gp150, gp140	CS (*n* = 9) RS (*n* = 5)
		T1	NYVAC + placebo	NYVAC + placebo	NYVAC + AIDSVAX® B/E	NYVAC + AIDSVAX® B/E	gp140, gp120	CS (*n* = 6) RS (*n* = 9) Saliva (*n* = 19) SP (*n* = 10)
	HVTN 096 NCT01799954	T2	NYVAC + AIDSVAX® B/E	NYVAC + AIDSVAX® B/E	NYVAC + AIDSVAX® B/E	NYVAC + AIDSVAX® B/E	gp140, gp120	CS (*n* = 2) RS (*n* = 7) Saliva (*n* = 19) SP (*n* = 6)
		T3	DNA-HIV-PT123 + placebo	DNA-HIV-PT123 + placebo	NYVAC + AIDSVAX® B/E	NYVAC + AIDSVAX® B/E	gp140, gp120	CS (*n* = 3) RS (*n* = 7) Saliva (*n* = 17) SP (*n* = 7)
		T4	DNA-HIV-PT123 + AIDSVAX® B/E	DNA-HIV-PT123 + AIDSVAX® B/E	NYVAC + AIDSVAX® B/E	NYVAC + AIDSVAX® B/E	gp140, gp120	CS (*n* = 6) RS (*n* = 9) Saliva (*n* = 19) SP (*n* = 5)
	HVTN 097 NCT02109354	T1^a^	ALVAC	ALVAC	ALVAC + AIDSVAX® B/E	ALVAC + AIDSVAX® B/E	gp120	CS (*n* = 7) RS (*n* = 18) SP (*n* = 10)
		T2	ALVAC	ALVAC	ALVAC + AIDSVAX® B/E	ALVAC + AIDSVAX® B/E	gp120	CS (*n* = 4) RS (*n* = 6) SP (*n* = 4)
Protein only	HVTN 088 NCT01376726	T1^b^	gp140/MF59	gp140/MF59	gp140/MF59	–	gp140^b^	CS (*n* = 5) RS (*n* = 10) Saliva (*n* = 16) SP (*n* = 7)
		T2	gp140/MF59	gp140/MF59	gp140/MF59	–	gp140	CS (*n* = 1) RS (*n* = 10) Saliva (*n* = 20) SP (*n* = 10)
Vector only	HVTN 205 NCT00820846	T4	MVA	MVA	Placebo	MVA	gp150	CS (*n* = 11) RS (*n* = 7) SP (*n* = 27)

Immunization regimens are shown for HVTN 076, 088, 086, 096, 097, and 205. Vaccine regimens were classified into four types (1–4), as indicated.

*CS* cervical sponge, *RS* rectal sponge, *SP* seminal plasma.

^a^Participants in HVTN 097 group T1 received also received Tetanus toxoid vaccine (Tetavax®) 1 month prior to initiation of the HIV vaccine administration schedule, and Hepatitis B vaccine (ENGERIX-B®) 2 weeks following the final HIV vaccine administration.

^b^Participants in HVTN 088 group T1 (primed) include previously vaccinated participants from HVTN 049 who received a DNA prime + gp140 protein boost, and participants from AVEG trials who received a vector prime and vector + gp120 protein boost. For the analyses in this manuscript, all previously vaccinated participants are categorized in the protein boost the only group, due to the long rest interval between immunizations.

### Protein immunization increased mucosal HIV-1-specific IgG antibodies

**Fig. 1:**
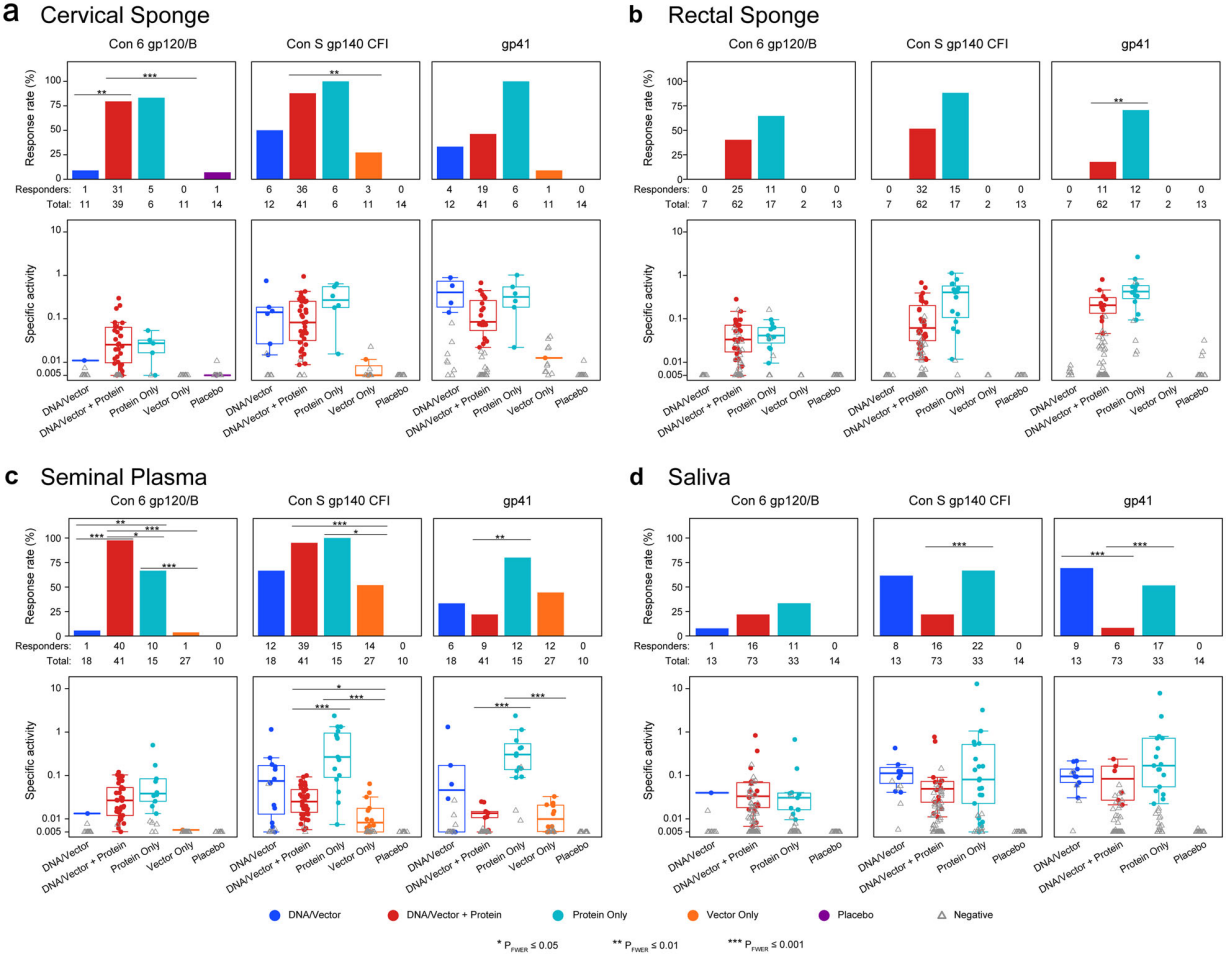
Protein immunization elicits a high response rate of mucosal HIV-1 gp120 envelope-specific IgG. HIV-1 envelope-specific IgG (Con 6 gp120, Con S gp140, and gp41) were measured in secretions from **a** cervical sponge, **b** rectal sponge, **c** seminal plasma, and **d** saliva. The antibody magnitude was calculated as the HIV-specific concentration relative to total antibody concentration, noted as specific activity (SA = MFI*dilution/total IgG ng per mL). Top panels represent the response rates and bottom panels represent the response magnitudes (solid dots for positive responses and grey open triangles for negative responses). In the bottom panels, the mid-line of the boxplot denotes the median response magnitude and the ends of the boxplot denote the 25th and 75th percentiles among positive responses. Differences in the response rate between vaccine regimens were assessed using Barnard’s exact test and differences in response magnitude were assessed using the Wilcoxon rank-sum test. *P_FWER_ < 0.05, **P_FWER_ < 0.01, and ***P_FWER_ < 0.001.

Robust levels of vaccine-elicited serum IgG responses were described for each regimen in this meta-analysis^19,21–24^ (Supplementary Table 2). Since mucosal surfaces are the primary sites of HIV-1 exposure and infection, we further investigated the presence and magnitude of antibody responses elicited in mucosal compartments for each broad vaccine type. The assay exhibited excellent specificity for vaccine-elicited responses, with a <1% false-positive rate for both IgG (1/153 measurements) and IgA (1/64 measurements) in placebo recipients. We found that IgG binding antibodies to gp41 and gp140 were elicited by all vaccine types (Supplementary Tables 1) in cervical secretions (Fig. 1a), in seminal plasma (Fig. 1c), and in saliva (except the Vector Only vaccine trial, which did not collect saliva samples) (Fig. 1d), although response frequency and magnitudes to gp140 and gp41 in cervical secretions were low among participants receiving Vector Only vaccinations, and response frequency to gp140 and gp41 in saliva was also low among those receiving DNA/vector + protein vaccinations. In contrast, vaccine-elicited gp41 and gp140 IgG in rectal secretions (Fig. 1b) were only observed in participants receiving a protein boost (DNA/vector + protein or protein only). In addition, vaccine regimens containing a protein boost exhibited the highest response rates to all antigens in seminal plasma and in cervical and rectal secretions vs. DNA/vector or vector-only regimens (Fig. 1a–c). Total elicited IgG and total IgA were similar among the vaccine types within the different mucosal compartments (Supplementary Fig. 1, see overlapping colored dots). Anti-gp120 and gp140 IgG response rates in saliva were generally lower than in other mucosal compartments for DNA/vector, DNA/vector + protein, and protein only vaccine types (Fig. 1d).

### HIV-1 specific mucosal IgA can be induced at low levels and low frequency in vaccination

**Fig. 2:**
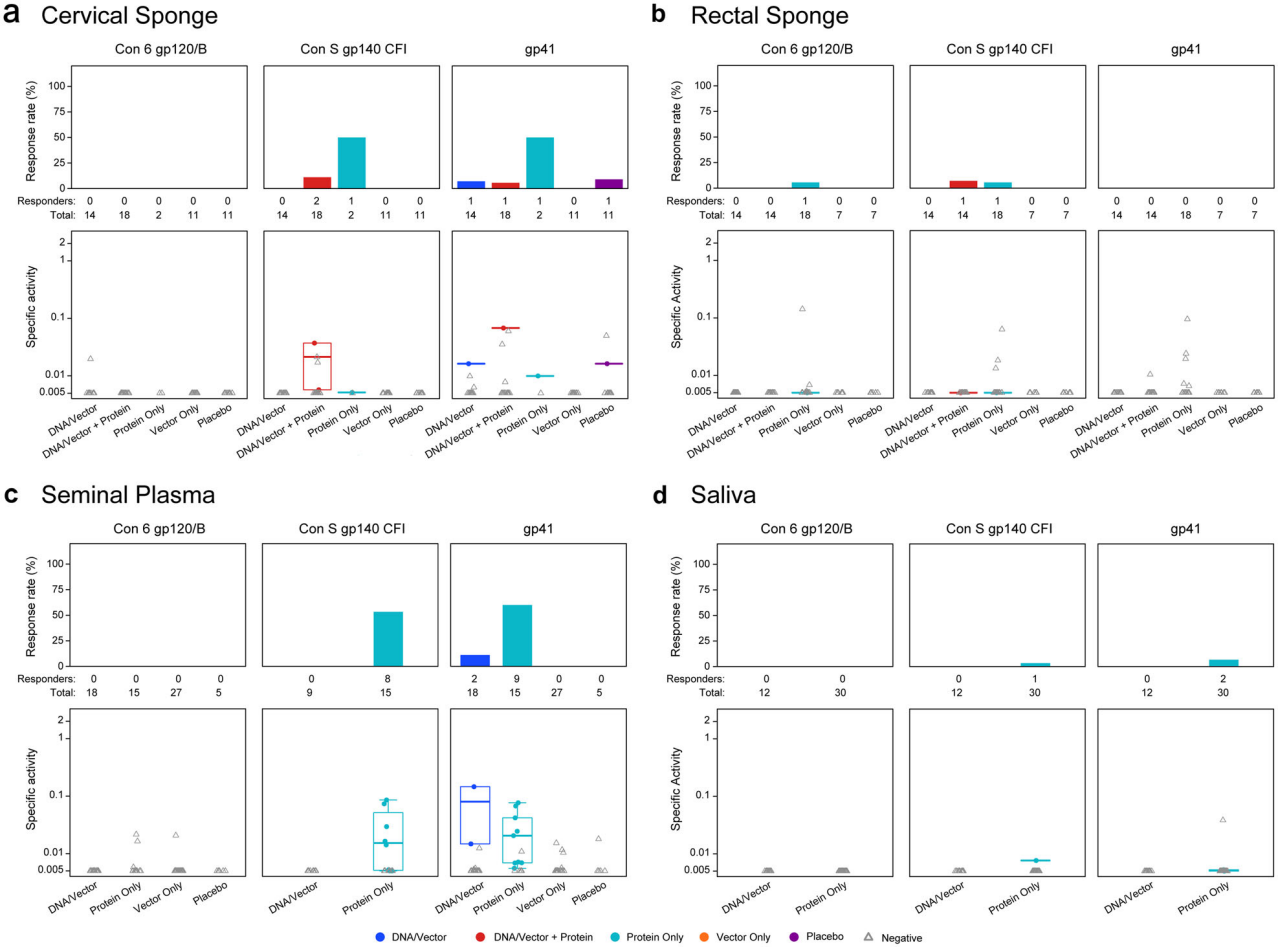
Env-specific IgA is rarely elicited in vaccinees and is predominantly to gp41. HIV-1 envelope-specific IgA (Con 6 gp120, Con S gp140, and gp41) responses were measured in secretions from **a** cervical sponge, **b** rectal sponge, **c** seminal plasma, and **d** saliva. The antibody magnitude was calculated as the HIV-specific concentration relative to total antibody concentration noted as specific activity (SA = MFI*dilution/total IgA ng per mL). Top panels represent the response rates and bottom panels represent the response magnitudes (solid dots for positive responses and grey open triangles for negative responses). In the bottom panels, the mid-line of the boxplot denotes the median response magnitude and the ends of the boxplot denote the 25th and 75th percentiles among positive responses.

IgA is abundant in mucosal secretions and HIV-1 specific IgA is modestly elicited post-HIV-1 infection^25–29^. However, the functional role of HIV-1-specific IgA in the prevention of HIV-1 infection remains unclear^25,30–34^. Although mucosal IgG was readily elicited across vaccine regimens and mucosal compartments, vaccine-elicited HIV-1-specific mucosal IgA was infrequently observed and of low magnitude (Fig. 2, Supplementary Table 1). The highest IgA response rates were against gp140 and gp41 antigens in seminal plasma from previously HIV-1 vaccinated participants who received a clade C gp140/MF59 boost in HVTN 088 (protein only, clade C gp140/MF59, Table 1) 6–17 years following the last vaccination. This suggests that the detection of HIV-1 specific IgA may be due to the additional late boost after a long rest period. As seen with IgG, total IgA recovered was similar among vaccine regimens within mucosal compartments (Supplementary Fig. 1A).

### gp41 and gp140 antibodies dominated the mucosal response

We previously reported that gp160, gp150, and gp140 immunogens elicit greater systemic antibody response rates and binding antibody magnitude to gp41 and gp140 compared to gp120 antigens^35–37^. To determine whether anti-gp41 and anti-gp140 responses were also preferentially elicited or localized to mucosal compartments across vaccine types, we examined mucosal antibody responses for those regimens that contained gp140, gp150, or gp160 sequences in the boost. Notably, in all mucosal compartments, anti-gp140 response rates were significantly higher than response rates to gp41 and gp120 (Table 2, *P*_FWER_ < 0.01). Magnitudes of IgG responses against gp140 were also significantly higher than the IgG magnitudes against gp120 in all mucosal compartments and the IgG magnitudes against gp41 in seminal plasma (Table 3, *P*_FWER_ < 0.0001). gp41 IgG response magnitude (Table 3) was also significantly higher than gp120 IgG response magnitude in saliva, rectal secretions, and cervical secretions (*P*_FWER_ < 0.01), indicating potential differential elicitation or distribution to mucosal tissues.

**Table 2:** Mucosal anti-gp140 response rates are significantly increased vs. anti-gp120 and gp41 responses in all mucosal compartments.

	Rate (%)	*P*-value	*P*_FWER_^a^
gp120 vs. gp140			
Cervical sponge	48 vs. 71	**0.0001**	0.0010***
Rectal sponge	33 vs. 52	**0.0002**	0.0015**
Saliva	24 vs. 39	**<0.0001**	0.0005***
Seminal plasma	44 vs. 76	**<0.0001**	<0.0001***
gp120 vs. gp41			
Cervical sponge	48 vs. 51	0.8145	>0.9999
Rectal sponge	33 vs. 33	>0.999	>0.9999
Saliva	24 vs. 27	0.4328	>0.9999
Seminal plasma	44 vs. 44	>0.9999	>0.9999
gp140 vs. gp41			
Cervical sponge	71 vs. 51	**0.0005**	0.0024**
Rectal sponge	52 vs. 33	**0.0002**	0.0015**
Saliva	39 vs. 27	**0.0017**	0.0070**
Seminal plasma	76 vs. 44	**<0.0001**	<0.0001***

Bold values are statistically significant.

*P_FWER_ ≤ 0.05; **P_FWER_ ≤ 0.01; ***P_FWER_ ≤ 0.001.

^a^P_FWER_ is the adjusted *P*-value for multiple comparisons to control the family-wide type I error rate.

**Table 3:** Mucosal anti-gp41 and anti-gp140 IgG response magnitude is significantly increased vs. anti-gp120 IgG in cervical, rectal and oral secretions.

	Median^a^	*P*-value	*P*_FWER_^b^
gp120 vs. gp140			
Cervical sponge	0.008 vs. 0.126	**<0.0001**	<0.0001***
Rectal sponge	0.017 vs. 0.149	**<0.0001**	<0.0001***
Saliva	0.016 vs. 0.069	**<0.0001**	<0.0001***
Seminal plasma	0.009 vs. 0.024	**<0.0001**	<0.0001***
gp120 vs. gp41			
Cervical sponge	0.009 vs. 0.080	**<0.0001**	<0.0001***
Rectal sponge	0.021 vs. 0.248	**<0.0001**	<0.0001***
Saliva	0.016 vs. 0.068	**0.0013**	0.0065**
Seminal plasma	0.012 vs. 0.007	0.1323	0.3969
gp140 vs. gp41			
Cervical sponge	0.126 vs. 0.074	0.8683	0.8683
Rectal sponge	0.149 vs. 0.189	0.4994	0.8683
Saliva	0.069 vs. 0.055	**0.0126**	0.0506
Seminal plasma	0.024 vs. 0.007	**<0.0001**	<0.0001***

Bold values are statistically significant.

*P_FWER_ ≤ 0.05; **P_FWER_ ≤ 0.01; ***P_FWER_ ≤ 0.001.

^a^Median among the participants who had a positive response to at least one antigen.

^b^*P*_FWER_ is the adjusted *P*-value for multiple comparisons to control the family-wide type I error rate.

### Persistent low concentrations of mucosal IgG boosted after a prolonged rest period

**Fig. 3:**
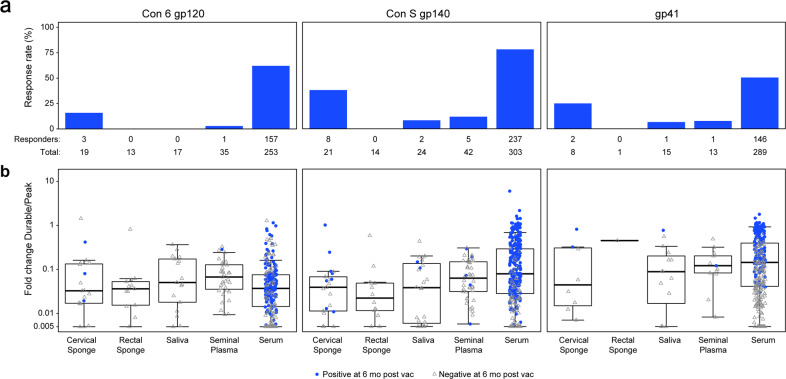
Rapid decline in Mucosal Env-specific IgG responses 6 months post-last vaccination from the peak. **a** The response rate for detectable HIV-1 envelope-specific IgG (Con 6 gp120, Con S gp140, and gp41) in the mucosal secretions and serum at 6 months post-the last vaccination by mucosal compartment. **b** The fold-change in the magnitude of HIV-1 envelope-specific IgG (Con 6 gp120, Con S gp140, and gp41) from the measured peak immune responses to 6 months following the last vaccination (SA durability timepoint/SA peak time point) was determined for each mucosal compartment and serum. A fold-change of 1 indicates no decline in mucosal response magnitude at 6 months from the peak. Blue circles indicate positive vaccine responders at 6 months post-last vaccination; and open gray triangles indicate negative vaccine responders at 6 months post-last vaccination.

Elicitation of long-lived antibodies post-vaccination, particularly in mucosal compartments, is a key goal for HIV-1 vaccine development. The durability of serum antibody responses to specific HIV antigens is variable, with half-life estimates for vaccine-elicited IgG in RV144, for example, ranging from 11.7 to 23.7 weeks^38^. Elicitation of HIV-1-specific mucosal antibodies through vaccination in humans has been reported previously^39,40^ as well as in the current study; however, long-term durability for vaccine-induced mucosal antibodies has not been reported to date. We tested genital, rectal, and oral secretions for the presence of HIV-1-specific antibodies 6 months post-last vaccination in three trials: HVTN 076 (Vector only, DNA/Ad5 regimen), 096 (DNA/Vector + protein, NYVAC or DNA/AIDSVAX B/E regimen) and 097 (DNA/vector + protein, ALVAC/AIDSVAX B/E regimen) and compared elicited mucosal response rates and fold change (between the durability and peak timepoints) at mucosal sites with that of vaccine-elicited serum antibodies. Low-level but persistent mucosal gp41-, gp140-, and gp120-specific IgG antibodies were present in up to 38% of vaccinees 6 months post-last vaccination, predominantly Con S gp140 IgG in cervical secretions (Fig. 3a). In contrast, serum antibody responses were present in up to 78% of participants, and at higher levels than mucosal IgG 6 months post-last vaccination (Fig. 3a). Although formal analysis of fold-decline is limited due to mucosal sample availability and low mucosal response rates at durability timepoints, median fold-decline from peak to 6 months post-last vaccination was similar between mucosal and systemic compartments for all antigens tested. Median fold-change in binding magnitude across all compartments for Con 6 gp120 and Con S gp140 was >1 log from peak immunogenicity to the durability timepoint (Fig. 3b).

**Fig. 4:**
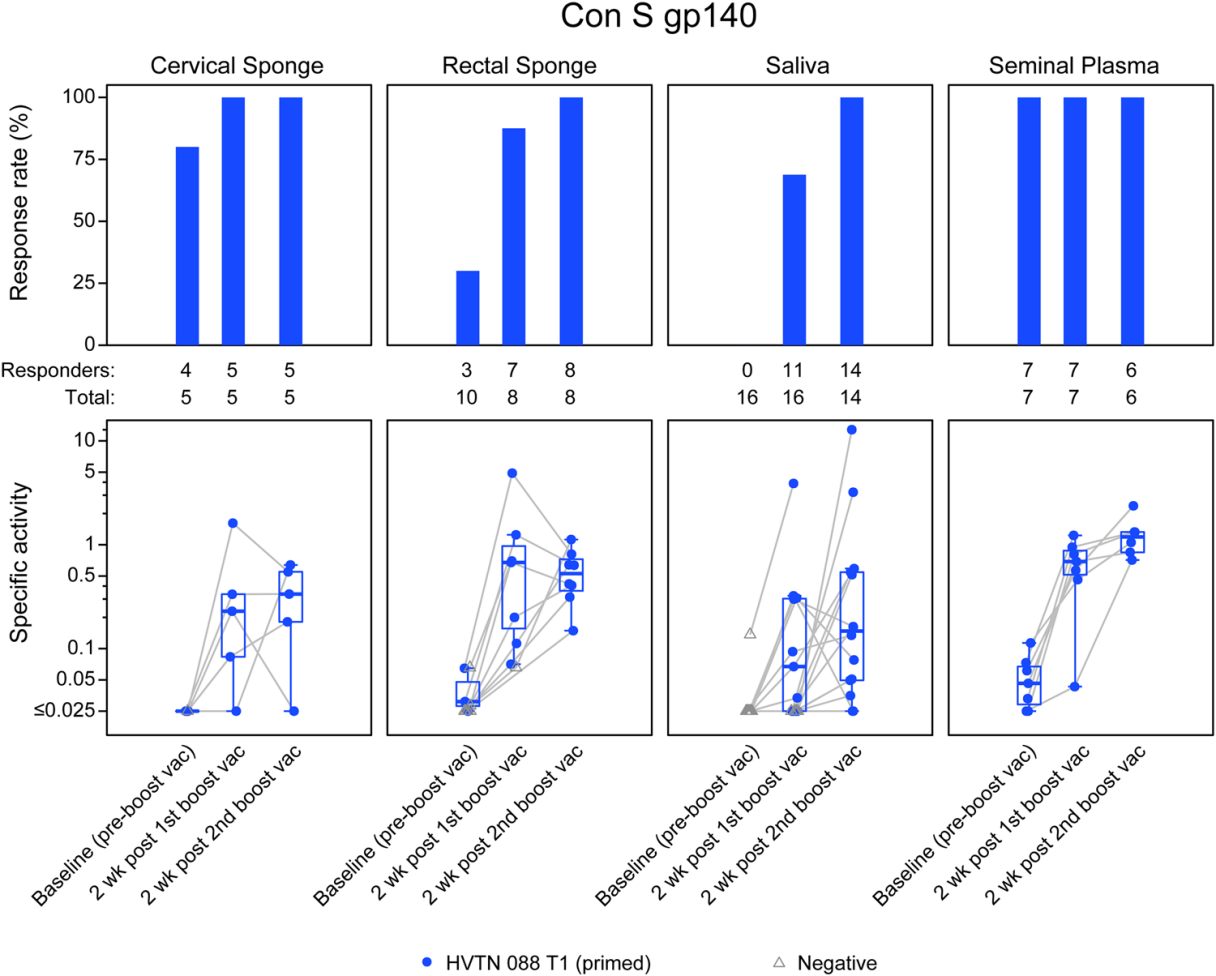
Persistent low concentrations of mucosal IgG boosted after a prolonged rest period. Antibodies were evaluated for persistence 6–17 years (baseline) post-last vaccination in the protein-only vaccine regimen, HVTN 088, given to previously immunized participants. The response rate for detectable HIV-1 Con S gp140-specific IgG was measured in cervical and rectal secretions, saliva, and seminal plasma at baseline (pre-boost) and 2 weeks after the first and second boost vaccinations (top panels). The concentration of HIV-1 Con S gp140-specific IgG was calculated as the HIV-specific concentration relative to total antibody concentration noted as specific activity (SA = MFI*dilution/total IgG ng per mL) (bottom panels). Blue circles indicate positive vaccine responders and open gray triangles represent non-responders at each timepoint. The boxplots are for positive responses (the mid-line of the boxplot denotes the median and the ends of the boxplot denote the 25th and 75th percentiles). The grey lines connect the observations between the timepoints.

We next investigated the long-term durability of mucosal antibodies and whether these antibodies can be detected after a prolonged rest period. Study participants previously vaccinated with either a DNA vaccine boosted by a subtype B gp140 vaccine with MF59 adjuvant (HVTN 049^41^) or with ALVAC-HIV vaccine boosted by a subtype B gp140 vaccine with MF59 adjuvant, were boosted with a heterologous Clade C vaccine 6–17 years post-last vaccination (HVTN 088^20^, Treatment group 1, T1). As shown in Fig. 4, HIV-1-specific IgG responses to Con S gp140 protein were present in cervical and rectal secretions in 30–100% of previously vaccinated participants at baseline (pre-boost vaccination, Fig. 4), demonstrating the feasibility of eliciting long-lived mucosal antibody responses with an HIV-1 vaccine. IgG antibody response rates in cervical secretions, seminal plasma, and rectal secretions were 80%,100%, and 30%, respectively, at baseline. Mucosal antibody responses were rapidly boosted with a heterologous gp140 protein boost, achieving 69–100% response rates after the first boost and a 100% response rate after the second boost in all mucosal compartments.

### Circulating HIV-1 IgG responses in the blood exhibited a range of correlation with mucosal IgG

**Fig. 5:**
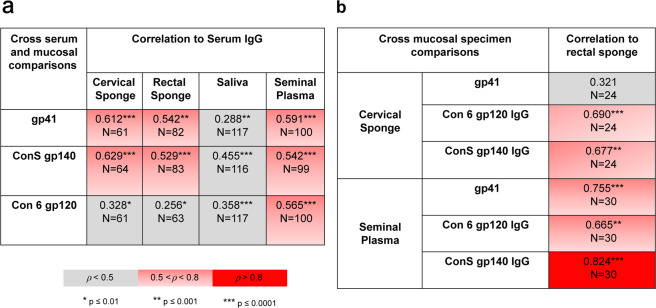
Circulating HIV-1 IgG in the blood exhibits a low-moderate correlation with mucosal IgG. Spearman correlation of IgG adjusted for vaccine-type (**a**) between mucosal compartments and serum, and (**b**) between rectal and cervical sponge for women and between a rectal sponge and seminal plasma for men. *, **, and *** indicates significance at *p* < 0.01, *p* < 0.001, and *p* < 0.0001; respectively.

Spearman correlations between mucosal Env IgG responses and serum Env IgG responses in 854 samples (476 serum, 101 seminal plasma, 119 saliva, 88 rectal, and 70 cervical) collected at peak immunogenicity timepoints were computed to determine whether mucosal antibody responses correlated with serum antibody responses after adjusting for vaccine type. Mucosal IgG responses exhibited a range of significant correlations with serum IgG responses (Fig. 5). Moderate, yet statistically significant correlations with serum IgG levels were observed for Con S gp140 and gp41 IgG responses in cervical and rectal secretions, and for Con S gp140, gp41, and Con 6 gp120 in seminal plasma (Spearman Correlation rho > 0.5, *p* < 0.001). Saliva IgG responses correlated poorly with serum IgG responses (rho < 0.5) for all Env epitope specificities. Con 6 gp120 responses also correlated poorly with serum IgG responses in cervical and rectal secretions (rho < 0.5).

We next examined whether responses in rectal secretions correlated with responses in cervical secretions within female participants or with seminal plasma within male participants. In males gp140 antibody responses in seminal plasma were strongly correlated with responses in rectal secretions (rho > 0.8, *p* < 0.001), while gp41 and gp120 responses between rectal and seminal compartments were modestly correlated (rho = 0.755, *p* < 0.0001 and 0.665, *p* < 0.001, respectively) (Fig. 5b). In contrast, in females, cervical gp41 responses were not correlated with rectal gp41 responses (rho = 0.321, *p* = 0.13), while moderate correlations were observed for gp120 and gp140 IgG responses between cervical and rectal secretions (rho < 0.7, *p* < 0.01), indicating potential gender-specific differences in antibody distribution in the rectum and genital tract.

## Discussion

The induction of HIV-1-specific humoral responses in the mucosa by vaccine immunogens is critical for both bnAb and non-bnAb vaccine development strategies. Protective immunity at these portals of entry could block both HIV-1 acquisition and replication. Understanding the concentrations and specificities of antibodies elicited by the diverse immunogen strategies of DNA, viral vector, and protein vaccines provide a framework for designing and assessing future vaccine regimens that target particular specificities and known effective concentrations for a protective effect. Here, we examined the presence, isotype, and epitope specificities of vaccine-elicited antibodies across multiple HIV-1 vaccine regimens, categorized as DNA/vector, DNA/vector + protein, vector-only, and protein only immunization strategies.

This meta-analysis led to three important findings with implications for HIV-1 vaccine design. First, we determined that vaccine regimens containing a protein component exhibit significantly higher IgG response rates to HIV-1 Env antigens in seminal plasma, and in cervical and rectal secretions and higher mucosal IgA responses in seminal plasma than regimens containing DNA/vector or vector only. Second, the correlation of serum and mucosal IgG responses depends on specimen type and antibody specificity. Most notable was that gp41 IgG responses poorly correlated between the cervical and rectal compartments in females and that gp120 IgG in both of these compartments poorly correlated with the levels of circulating IgG in serum. Third, mucosal antibody responses can be durable and boosted with subsequent immunization, similar to boosting observed in serum upon re-vaccination. These results indicate that vaccine-elicited systemic IgG responses provide an incomplete picture of anti-viral antibodies at mucosal surfaces in females, which may be differentially influenced by factors such as hormonal levels and the local microenvironment. Therefore, the identification of mucosal antibody immune correlates of protection may require mucosal sampling in vaccine efficacy trials.

Our work is consistent with previous findings that systemic vaccination regimens can elicit IgG-specific mucosal responses^39,40,42^, and that elicitation of mucosal HIV-1-specific IgA is rarely observed in systemic vaccination strategies and IgA responses are of low magnitude when present^40,43–45^). Further testing of vaccine strategies with adjuvants that differentially stimulate the innate response is needed to improve mucosal IgA responses for vaccines administered intramuscularly. One promising result from a rhesus macaque vaccine study suggests that immunization of HIV Env immunogens with GLA + 3M052 adjuvant can stimulate the highest magnitude and breadth of mucosal IgA responses when compared to other adjuvants (Bali Pulendran, Stanford University, personal communication). Mucosal vaccination strategies may improve the elicitation of potentially protective IgA at mucosal sites^46–49^. Elicitation of high levels of certain Env-specific IgA in serum correlated with decreased vaccine efficacy in the RV144 trial^30^ and also modulated the association of antibody Fc effector functions with decreased HIV-1 risk in HVTN 505^50^. However, the role of mucosal IgA in vaccine efficacy was not determined as mucosal samples were not collected in these clinical trials. There is evidence from preclinical rhesus macaque protection studies, results from highly exposed seronegative cohorts, and in vitro studies of the antiviral properties of IgA monoclonals and that support a potentially protective role for IgA in the mucosa^14,16,25,27,51,52^.

We also found that similar to serum IgG responses elicited by these regimens^19,35–37^, vaccine regimens containing a gp160, gp150, or gp140 component preferentially elicited mucosal gp41 specificities with the HIV envelope antigens tested here. Previous findings raise the hypothesis that some gp41 specific antibody responses may originate from cross-reactive antibodies to the intestinal microbiome and that vaccine-elicited antibodies can correlate with the gut microbiota composition^35,53^. The elicitation of gp41 mucosal specificities parallels that of serum; however, further investigation is needed to understand any potential impact of local microbial microenvironments in the modulation of mucosal immune responses. In vaccine regimens utilizing three components (DNA, viral vector, protein), we observed that rectal IgG responses (in participants from HVTN 088, 096, and 097) were more strongly correlated with seminal plasma IgG responses compared to cervical responses (from participants in HVTN 086, 088, 096, 097, and 205) in male and female participants, respectively. The presence of HIV-1 envelope-specific IgA in 50% of the seminal plasma (*n* = 15) and cervical specimens (*n* = 2) in the protein-only boost immunization of the protein-only group (HVTN 088) is striking in comparison to the negligible response (0 to <15%) in the other vaccine regimens. This finding indicates the importance of the protein component of the vaccine, and/or a boost after a long rest period if genital tract IgA immunity is found to correlate with protection. This is especially noteworthy considering that in seminal plasma, IgG and not IgA is the more dominant of the two isotypes^54^. Furthermore, these studies highlight sex-specific differences in antibody levels in mucosal compartments, along with a lower correlation between antibody levels in cervical and rectal compartments in females vs. seminal plasma and rectal compartments in males. Thus, additional investigation is needed into potential sex-specific differences in antibody trafficking to genital compartments and how different vaccine regimens may influence antibody induction. Potential sex-specific differences may be due, in part, to known factors regulating antibody responses at mucosal sites, including: (1) hormonal regulation of immunoglobulin levels in females (2) presence or absence of concurrent STIs, and (3) the predominance of IgA and secretory IgA in intestinal mucosal vs. IgG predominance in the male and female genital tracts.

A major barrier to the development of an efficacious HIV-1 vaccine is the capacity to induce long-lasting antibody responses with the right specificity, function, and magnitude at the mucosa to mediate protection. We found that up to 38% of participants maintained vaccine-elicited binding antibodies in cervical secretions 6 months post-last vaccination, and fold change from durability timepoints to the peak was similar between serum and mucosal compartments. In addition, antibody decline (fold-change) was similar between vaccine responders and non-responders at 6 months post-last vaccination, indicating that successful vaccines will need to induce sufficient levels of mucosal antibodies at a peak in order to maintain potentially protective responses at mucosal portals of entry. In a trial designed to evaluate antibodies after long-term follow-up and boost after a long rest period (HVTN 088), we evaluated the durability of vaccine-elicited cervical and rectal antibodies at time points from 6 months up to 17 years post-vaccination. We observed remarkable durability of envelope-specific gp140 antibody responses with the capacity to be boosted to higher concentrations compared to vaccine-naïve individuals. These data provide a proof-of-concept that mucosal antibody responses can be boosted after a long duration and may provide increased levels of the antibody with a delayed boost. Since robust antibody concentrations were detected in mucosal fluids, it will be important to determine if these concentrations are sufficient for protection and to define vaccine immunogens that can induce the optimal antibody specificities.

Limitations of the current analyses include the lack of availability of paired rectal and seminal samples for all but the HVTN 088 (protein only, gp140/MF59), 096 (DNA/vector + protein, NYVAC or DNA/AIDSVAX B/E), and 097 (DNA/vector + protein, ALVAC/AIDSVAX B/E) vaccine regimens, limitation of these analyses to only regimens with a gp140, gp150, or gp160 component, and sample collection which was limited to peak serum immunogenicity timepoints. We also recognize that the findings from this work are specific to the clinical trials studied here and may be limited to the specific study populations, the specific immunogens, and adjuvants, and collection methods tested. Future analyses are also needed to assess the impact of concurrent STIs on vaccine-induced antibody responses and to continue to evaluate studies that compare different delivery routes of immunization, such as subcutaneous^55^ and mucosal^56–58^ with the next-generation vaccine immunogens. In addition, previous work in HIV-1 infected individuals demonstrated the presence of anti-HIV antibodies in the female genital tract with either neutralizing^2^ or non-neutralizing effector functions^1,59,60^, indicating proof-of-concept that functional antibodies can be elicited at mucosal sites. Future studies profiling mucosal antibody Fc effector functions and subclass specificity may provide further clues to potential mechanisms of mucosal protection in vaccine studies. Several HIV vaccine clinical trials that are ongoing or recently completed have collected mucosal samples and analyses from these trials will be important to extend the findings of this cross-protocol analysis.

In conclusion, we have shown that HIV-1 vaccine regimens with a protein component enhance mucosal antibody response rates and that vaccines containing a gp140 component preferentially elicit gp41 antibodies over gp120 antibodies. HIV-1 gp120 responses were of relatively low magnitude and weakly correlated with serum responses in cervical and rectal secretions. Further work is needed to evaluate the impact of different vaccine regimens (e.g., vector, gp120 protein, adjuvant) on the magnitude, specificity, and functions of mucosal antibody responses, which will advance HIV-1 vaccine development.

## Methods

### Human clinical trials and mucosal sampling

The HIV-1 vaccine clinical trials (HVTN 076: NCT00955006; HVTN 086: NCT01418235; HVTN 088: NCT01376726; HVTN 096: NCT01799954; HVTN 097: NCT02109354; and HVTN 205: NCT00820846) enrolled healthy HIV-1-negative participants assessed to be at low risk for HIV-1 acquisition. Optional mucosal sampling was performed as part of the exploratory objectives. All participants provided written informed consent prior to their enrollment. Serum/plasma and mucosal antibody responses to vaccination were evaluated at peak immunogenicity, which was 2 weeks post-last immunization for all studies except HVTN 076, where collections occurred 4 weeks post-last immunization. Cervical, rectal, saliva, seminal plasma, and rectal biopsies were available for this study from HVTN 076, HVTN 086, HVTN 088, HVTN 096, HVTN 097, and HVTN 205 (Table 1).

The following Ethics or Institutional Review Boards (IRB) reviewed and approved the following protocols: Fred Hutchinson Cancer Research Center IRB (HVTN 076, 088, 205), University of KwaZulu-Natal Biomedical Research Ethics Committee (HVTN 086), University of Witwatersrand Human Research Ethics Committee (HVTN 086, 097), University of Cape Town Human Research Ethics Committee (HVTN 086, 097), Vanderbilt University IRB (HVTN 088, 205), University of Rochester IRB (HVTN 088, 205), the University of Alabama at Birmingham IRB (HVTN 088, 205), Canton de Vaude Commission Cantonale d’ethique de la recherce (HVTN 096), Emory University IRB (HVTN 205), New York Blood Center IRB (HVTN 205), Fenway Community Health IRB (HVTN 205), Columbia University Medical Center IRB (HVTN 205), Partners Human Research Committee (HVTN 205), UCSF Committee on Human Research, (HVTN 205), Comite Institucional de Bio-Etica Asociacion Civil IMPACTA Salud y Educacion (HVTN 205).

### Specimen collection procedures

Cervical secretions were collected using Merocel Sponge points (Beaver-Vistec; HVTN 076, HVTN 097) or Weck-Cel sponge points (Beaver-Vistec, HVTN 086, HVTN 088, HVTN 096, HVTN 205) by trained clinicians. Secretions were collected by holding the sponge point in or as close as possible to the cervical os until the sponge appeared saturated, or up to 2 min. The sponge was withdrawn and immediately placed into a cryovial, snap-frozen on dry-ice and stored at −80 °C until further use.

Rectal secretions were collected using Weck-Cel sponge spears (Beaver-Vistec, HVTN 086, HVTN 088, HVTN 096, HVTN 205) or Merocel sponge spears (Beaver-Vistec, HVTN 097) pre-moistened with Dulbecco’s Phosphate Buffered Saline (DPBS). A disposable anoscope was lightly lubricated on the obturator end of the anoscope and inserted into the rectum 7–8 cm. A moistened sponge spear was inserted into the anoscope which was withdrawn 2–3 cm to allow the sponge to be surrounded by rectal mucosa. The sponge was left in place for 5 min and then withdrawn and immediately frozen in a cryovial on dry-ice and stored at −65 °C to −90 °C until further use.

Seminal plasma was collected as follows. Participants were instructed to refrain from ejaculation for at least 48 h prior to semen collection. The glans were cleansed with a towelette prior to ejaculation and semen was collected directly into a specimen collection container without the use of lubricants other than water. Semen samples were kept on ice for up to 2 h for transport to the processing laboratory, where 2.5 mL of viral transport media were added to each specimen collection container. Samples were then centrifuged and the supernatant was aliquoted and subsequently stored at −65 °C to −90 °C until further use.

Saliva was collected as follows. Participants were instructed to avoid smoking, eating, or drinking anything but water for 1 h before collection. Participants chewed dental wax for up to 5 min to stimulate saliva production, then collected 3–5 mL of saliva by spitting it into a 50 mL sterile tube embedded in a cup of wet ice. Samples were transported on wet ice to the processing laboratory, centrifuged and the supernatant was aliquoted and subsequently stored at −65 °C to −90 °C until further use.

### HIV-1 mucosal antibody measurements

Measurements of HIV-1-specific IgG and IgA were performed under good clinical laboratory practices as previously described^26,27,61^. Briefly, HIV-1 proteins were conjugated to polystyrene beads (Luminex Corporation) and binding of IgG or IgA in mucosal secretions to the bead-conjugated HIV-1 proteins were detected by mouse anti-human IgG-Biotin (Southern Biotech) followed by Streptavidin-PE or goat anti-human IgA-Biotin (Jackson Immunoresearch) followed by Streptavidin-PE. For the detection of IgA responses, samples were first depleted of IgG using Protein G high-performance MultiTrap plates (General Electric) according to the manufacturer’s instructions. Sample binding intensities (MFI, Mean Fluorescence Intensities) were acquired on a Bio-Plex 200 instrument (Bio-Rad) using 21CFR Part 11 compliant software. Standard positive and negative controls were included in each assay to ensure specificity and for maintaining consistency and reproducibility between assays. 7B2 IgA monoclonal antibody (mAb) controls were kindly provided by Drs. Huaxin-Liao and Barton Haynes, Duke University. The preset assay criteria for sample reporting are: (1) coefficient of variation per duplicate values for each sample of <15% and (2) >100 beads counted per sample. To control for Env protein performance, a preset criterion is established that the positive control titer (HIVIG) included on each assay must be within ±3 standard deviations of the mean for each antigen (tracked with a Levey-Jennings plot with preset acceptance of titer and calculated with a four-parameter logistic equation, SigmaPlot, Systat Software).

Total IgG and IgA antibody measurements for calculating specific activity were performed using a Bio-Plex Pro Human Isotyping Kit (Bio-Rad) according to the manufacturer’s instructions. Ig concentrations were determined by 4-parameter logistic (4-PL) regression using the Bio-Plex Manager 6.0 software (Bio-Rad). IgG responses were measured at a 1:2 dilution in all protocols except HVTN 088, in which IgG responses were measured with multiple dilutions 1:2, 1:5, and 1:10 in cervical and rectal samples but with a single dilution 1:3 in saliva samples and 1:10 in seminal samples. We applied the four-parameter paired response curves (Fong, Permar and Tomaras “Four-parameter paired response curve for serial dilution assays”, JRSS Series C, in press) to build the model based on the IgG responses in cervical and rectal samples with 1:2 and 1:10 dilutions to predict the IgG responses at a 1:2 dilution from the IgG responses with 1:3 dilution in saliva and 1:10 dilution in seminal samples.

HIV-1-specific binding MFI values were normalized to total IgG or IgA and computed as specific activity (antigen-specific MFI*dilution factor/ng ml^−1^ total IgG or IgA). Samples were excluded from analysis if total IgG <48 ng/ml or if total IgA ≤0 ng/ml. Positivity calls per HIV-1 antigen per antibody isotype were based on three preset criteria determined as (1) sample MFI greater than the 95th percentile of baseline unvaccinated samples from all protocols in this analysis, per sample type, antigen, isotype, and detection method or 100, whichever is greater, (2) specific activity (SA) greater than three times the 95th percentile of all baseline unvaccinated samples from all protocols in this analysis, per sample type, antigen, isotype, and detection method, and (3) SA greater than three-times the subject-specific baseline when present. For the samples from HVTN 088 T1 primed group or the samples with missing the baseline SA, the median of all available baseline SA for a given antigen, isotype, and detection method was used to place the subject-specific baseline in the criterion (3). HIV-1-specific antibody response was considered positive if it met these positivity criteria.

### Statistical analyses

#### Comparison between vaccine regimens

Differences in response rates between vaccine regimens were assessed using Barnard’s test and differences in response magnitudes among positive responders between vaccine regimens were assessed using the Wilcoxon rank-sum test. The comparisons of response rates between vaccine types were only performed if there were data from at least ten samples per vaccine type and the comparisons of response magnitudes were only performed if there were at least five samples with positive responses per vaccine type. All comparisons were done by antigen and mucosal compartment. The cervical IgG response rate in the protein only vaccine regimen was not compared with response rates in other vaccine regimens due to <10 samples in the protein only regimen. All statistical tests were two-sided. For response rates, there are 39 comparisons in total. For magnitudes, there are 28 comparisons in total. The *p*-values were adjusted for multiple comparisons to control the family-wise error rate within each response type (rate and magnitude). The adjusted *p*-value is denoted by *P*_FWER_.

#### Comparison between antigens

Differences in response rates between antigens were assessed using McNemar’s test and differences in response magnitudes among positive responders (to at least one antigen) between antigens were assessed using the Wilcoxon signed-rank test. The comparisons were made by mucosal compartment and pooling all samples within each mucosal compartment. There are 12 comparisons for each response rate and each magnitude. Any *P*_FWER_ ≤ 0.05 is considered significant. Adjusted Spearmen correlations between specimen types were calculated using the partial Spearman correlation adjusting for vaccine type^62^. Fold differences from peak immunogenicity to the durability timepoint were calculated using SA at each timepoint. The differences in total IgG/IgA among vaccine types within each mucosal compartment were assessed using the Kruskal-Wallis test. All comparisons were done using SAS version 9.4 (Copyright © 2013, SAS Institute Inc., Cary, NC, USA). Adjusted Spearman correlations were done using PResiduals R package in R version 3.6 (R Core Team (2013). R: A language and environment for statistical computing. R Foundation for Statistical Computing, Vienna, Austria. URL http://www.R-project.org/.).

### Reporting summary

Further information on research design is available in the Nature Research Reporting Summary linked to this article.

## Supplementary information

Supplemental Information

Reporting Summary

## Data Availability

All data generated or analyzed during this study are included in this published article (and its Supplementary Information files) and available upon request.
