# Supplementary material for: Meta-analysis of HIV-1 vaccine elicited mucosal antibodies in humans
Source: NPJ Vaccines. 2021 Apr 15;6:56. doi: 10.1038/s41541-021-00305-8 (PMC8050318; doi:10.1038/s41541-021-00305-8)
Supplement: Supplementary file 1 — Supplemental Information [file 41541_2021_305_MOESM1_ESM.pdf]

SUPPLEMENTARY MATERIAL

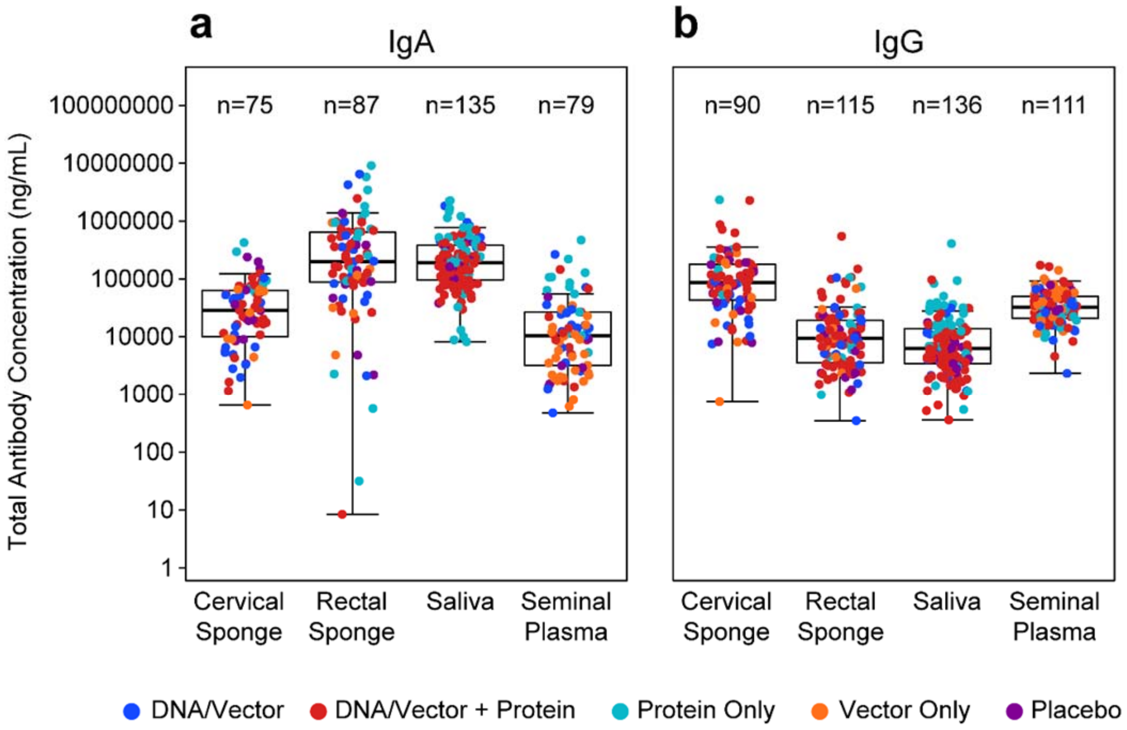

Supplementary Figure 1. Total Antibody concentrations. Boxplots of total IgA and IgG concentrations by mucosal compartment are shown in **A.** IgA and **B.** IgG. The mid-line of the boxplot denotes the median response magnitude and the ends of the boxplot denote the 25th and 75th percentiles. Solid circles represent the observations of total IgA and IgG, which were color-coded for vaccine regimen types and placebo.

Supplementary Table 1. Median Specific Activity Values by Vaccine Type and Mucosal compartment.

Isotype	Specimen Type	Vaccine Group	Median Response (SA)		
			Con 6 gp120/B	Con S gp140	gp41
IgA	Cervical Sponge	DNA/Vector	0.00025	0.00034	0.00113
		DNA/Vector+Protein	0.00020	0.00020	0.00137
		Placebo	0.00020	0.00020	0.00032
		Protein	0.00020	0.00267	0.00631
		Vector	0.00022	0.00020	0.00035
	Rectal Sponge	DNA/Vector	0.00020	0.00020	0.00055
		DNA/Vector+Protein	0.00020	0.00020	0.00113
		Placebo	0.00020	0.00020	0.00157
		Protein	0.00020	0.00020	0.00118
		Vector	0.00020	0.00020	0.00057
	Saliva	DNA/Vector	0.00020	0.00020	0.00021
		Protein	0.00020	0.00020	0.00020
	Semen	DNA/Vector	0.00020	0.00020	0.00120
		Placebo	0.00069	NA	0.00393
		Protein	0.00080	0.00235	0.00692
		Vector	0.00078	NA	0.00170
IgG	Cervical Sponge	DNA/Vector	0.00027	0.01519	0.02212
		DNA/Vector+Protein	0.01588	0.05361	0.01598
		Placebo	0.00020	0.00020	0.00053
		Protein	0.02189	0.26914	0.31871
		Vector	0.00030	0.00184	0.00896
	Rectal Sponge	DNA/Vector	0.00020	0.00036	0.00569
		DNA/Vector+Protein	0.01183	0.02628	0.00485
		Placebo	0.00056	0.00071	0.00146
		Protein	0.03526	0.31352	0.34506
		Vector	0.00020	0.00020	0.00020
	Saliva	DNA/Vector	0.00155	0.07355	0.09353
		DNA/Vector+Protein	0.01079	0.01336	0.00209
		Placebo	0.00048	0.00042	0.00069
		Protein	0.00305	0.02266	0.02233
	Semen	DNA/Vector	0.00023	0.01920	0.00390
		DNA/Vector+Protein	0.02649	0.02280	0.00282
		Placebo	0.00020	0.00020	0.00020
		Protein	0.02552	0.26628	0.15374
		Vector	0.00022	0.00469	0.00401

NA = Not applicable; SA values <0.0002 were set to a floor of 0.0002

Supplementary Table 2. Median Binding Magnitude (MFI) in Serum by Vaccine Type.

			Median Response (MFI)		
**Isotype**	**Specimen Type**	**Vaccine Group**	**Con 6 gp120/B**	**Con S gp140 CFI**	**gp41**
**IgA**	Serum	DNA/Vector	<100	<100	151
		DNA/Vector+Protein	285	308	141
		Placebo	<100	<100	<100
		Protein	<100	1324	2165
		Vector	<100	<100	137
**IgG**	Serum	DNA/Vector	149	4936	21881
		DNA/Vector+Protein	18584	23000	9578
		Placebo	<100	<100	172
		Protein	3567	23000	23000
		Vector	605	12760	23000

SUPPLEMENTARY ACKNOWLEDGEMENTS

HVTN 076 Study Team: Karen Mark[5], Janine Maenza[5], Ann Duerr[5], Cecilia Morgan[5], Stephen DeRosa[5], Gabriela Diaz[5], Niles Eaton[5], Edith Swann[6], Barney Graham[19]; HVTN 086 Study Team: Gavin Churchyard[8], Shelly Karuna[5], Peter Gilbert[5,15], Lynn Morris[20], Mary Allen[6], Carolyn Williamson[21], Anna-Lise Williamson[21], Susan Barnett[22]; HVTN 088 Study Team: Paul Spearman[7], Georgia Tomaras[1,2,3,4], Marnie Elizaga[5], Ying Huang[5, 15], Laissa Ouedraogo[6], Mary Allen[6], Susan Barnett[22]; HVTN 096 Study Team: Giuseppe Pantaleo[13], Pierre-Alexandre Bart[13], Shelly Karuna[5], Holly Janes[5], Shannon Grant[5], Nicole Frahm[5], Laissa Ouedraogo[6], Mary Allen[6], Song Ding[23], Edna Venneker[23], Carter Lee[24]; HVTN 097 Team: Glenda Gray[5,12], Surita Roux[5, 12], Fatima Laher[5, 12], Nicole Grunenberg[5], Ying Huang[5, 15], Abby Isaacs[5, 15], Carissa Karg[5], Ryan Jensen[5], Gina Escamilla[5], and Jill Zeller[5], Edith Swann[6], Georgia Tomaras[1,2,3,4], Sanjay Phogat[25], Carlos DiazGranados[25], Carter Lee[24], Faruk Sinangil[24]; HVTN 205 Team: Paul Goepfert[10], Kelly Seaton[1, 2, 3, 4], Marnie Elizaga[5], Alicia Sato[5], Laissa Ouedraogo[6], Julia Hutter[6],

Laura Polakowski[6], Harriett Robinson[14], HVTN Quality Assurance Team: Marcella Sarzotti-Kelsoe[26], Dan Ozaki[26], HVTN Operations Team: John Hural[5], On Ho[5], Michael Stirewalt[5], Mucosal Collections and Training Team: Philipp Mann[5], Craig Innes[5], Kathy Mngadi[27], Blythe Adamson[5], Alicia Yamamoto[5], Jenna Udren[5], Janine Maenza[5, 11], David Berger[5], Chris Galloway[5]

[19]Vaccine Research Center, National Institute of Allergy and Infectious Diseases, National Institutes of Health, Bethesda, Maryland, USA; [20]National Institute for Communicable Diseases, National Health Laboratory Services, Johannesburg, South Africa; [21]Institute of Infectious Disease and Molecular Medicine, University of Cape Town, South Africa; [22]Novartis Vaccines and Diagnostics, Cambridge, Massachusetts, USA; [23]EuroVacc Foundation, Lausanne, Switzerland; [24]Global Solutions for Infectious Diseases, South San Francisco, California, USA; [25]Sanofi Pasteur, Swiftwater, Pennsylvania, USA; [26]Quality Assurance for Vaccine Immunogenicity Programs, Duke University, Durham, NC, USA; [27]Tembisa Clinical Research Site, Johannesburg, South Africa
