# Supplementary material for: Meta-analysis of HIV-1 vaccine elicited mucosal antibodies in humans
Source: NPJ Vaccines. 2021 Apr 15;6:56. doi: 10.1038/s41541-021-00305-8 (PMC8050318; doi:10.1038/s41541-021-00305-8)
Supplement: Supplementary file 2 — Reporting Summary [file 41541_2021_305_MOESM2_ESM.pdf]

nature research

Corresponding author(s): Kelly E. Seaton

Last updated by author(s): Nov 19, 2020

Reporting Summary

Nature Research wishes to improve the reproducibility of the work that we publish. This form provides structure for consistency and transparency in reporting. For further information on Nature Research policies, see our Editorial Policies and the Editorial Policy Checklist.

Statistics

For all statistical analyses, confirm that the following items are present in the figure legend, table legend, main text, or Methods section.

n/a	Confirmed	
☐	☒	The exact sample size (*n*) for each experimental group/condition, given as a discrete number and unit of measurement
☐	☒	A statement on whether measurements were taken from distinct samples or whether the same sample was measured repeatedly
☐	☒	The statistical test(s) used AND whether they are one- or two-sided<br>*Only common tests should be described solely by name; describe more complex techniques in the Methods section.*
☒	☐	A description of all covariates tested
☐	☒	A description of any assumptions or corrections, such as tests of normality and adjustment for multiple comparisons
☒	☐	A full description of the statistical parameters including central tendency (e.g. means) or other basic estimates (e.g. regression coefficient) AND variation (e.g. standard deviation) or associated estimates of uncertainty (e.g. confidence intervals)
☐	☒	For null hypothesis testing, the test statistic (e.g. *F*, *t*, *r*) with confidence intervals, effect sizes, degrees of freedom and *P* value noted<br>*Give P values as exact values whenever suitable.*
☒	☐	For Bayesian analysis, information on the choice of priors and Markov chain Monte Carlo settings
☒	☐	For hierarchical and complex designs, identification of the appropriate level for tests and full reporting of outcomes
☐	☒	Estimates of effect sizes (e.g. Cohen's *d*, Pearson's *r*), indicating how they were calculated

Our web collection on statistics for biologists contains articles on many of the points above.

Software and code

Policy information about availability of computer code

Data collection	Binding Antibody Multiplex data were collected using a BioRad 200 machine and Bioplex Manager software, version 6.1, Security Edition.
Data analysis	SAS version 9.4 and R version 3.6

For manuscripts utilizing custom algorithms or software that are central to the research but not yet described in published literature, software must be made available to editors and reviewers. We strongly encourage code deposition in a community repository (e.g. GitHub). See the Nature Research guidelines for submitting code & software for further information.

Data

Policy information about availability of data

All manuscripts must include a data availability statement. This statement should provide the following information, where applicable:

- Accession codes, unique identifiers, or web links for publicly available datasets
- A list of figures that have associated raw data
- A description of any restrictions on data availability

All data generated or analyzed during this study are included in this published article (and its Supplementary Information files) and available upon request.

Field-specific reporting

Please select the one below that is the best fit for your research. If you are not sure, read the appropriate sections before making your selection.

☒ Life sciences ☐ Behavioural & social sciences ☐ Ecological, evolutionary & environmental sciences

For a reference copy of the document with all sections, see nature.com/documents/nr-reporting-summary-flat.pdf

Life sciences study design

All studies must disclose on these points even when the disclosure is negative.

Sample size	We included all available mucosal samples from previous HVTN clinical trials.
Data exclusions	We included all available data and didn't exclude any data in the analyses.
Replication	All samples were tested in duplicate using a validated Binding Antibody Multiplex Assay.
Randomization	All clinical trials described in this manuscript were randomized Phase I clinical trials.
Blinding	Investigators were blinded to group allocation during data collection and analysis.

Reporting for specific materials, systems and methods

We require information from authors about some types of materials, experimental systems and methods used in many studies. Here, indicate whether each material, system or method listed is relevant to your study. If you are not sure if a list item applies to your research, read the appropriate section before selecting a response.

Materials & experimental systems

n/a	Involved in the study	
☐	☒	Antibodies
☒	☐	Eukaryotic cell lines
☒	☐	Palaeontology and archaeology
☒	☐	Animals and other organisms
☐	☒	Human research participants
☐	☒	Clinical data
☒	☐	Dual use research of concern

Methods

n/a	Involved in the study	
☒	☐	ChIP-seq
☒	☐	Flow cytometry
☒	☐	MRI-based neuroimaging

Antibodies

Antibodies used	All monoclonal antibodies used as controls and all detection antibodies used in the custom Binding Antibody Multiplex Assay are referenced in the methods section of the manuscript. References for antibodies used in the BioRad Human Isotyping Kit may be found on the supplier's website.
Validation	Anti-human IgG (Southern Biotech) - https://www.southernbiotech.com/?catno=9042-08&type=Monoclonal#&panel1-2&panel2-1<br>Anti-human IgA (Jackson ImmunoResearch) - https://www.jacksonimmuno.com/catalog/products/109-065-011<br>BioRad Human Isotyping Kit - https://www.bio-rad.com/en-us/sku/171a3100m-bio-plex-pro-human-isotyping-panel-6-plex?ID=171A3100M

Human research participants

Policy information about studies involving human research participants

Population characteristics	Population characteristics for each clinical trial are described in the primary manuscript for each trial, referenced in the Results section, subheading "Meta-Analysis of human HIV-1 vaccine regimens includes DNA and vector prime with protein boost immunogens".
Recruitment	Participants were recruited into each study protocol through local clinical sites. Participants opted into collection of mucosal samples for each protocol as these sample types were not mandatory collections for protocols 086, 088, 097, 205.
Ethics oversight	The following Ethics or Institutional Review Boards (IRB) reviewed and approved the following protocols: Fred Hutchinson Cancer Research Center IRB (HVTN 076, 088, 205), University of KwaZulu-Natal Biomedical Research Ethics Committee (HVTN 086), University of Witwatersrand Human Research Ethics Committee (HVTN 086, 097), University of Cape Town Human Research Ethics Committee (HVTN 086, 097), Vanderbilt University IRB (HVTN 088, 205), University of Rochester IRB (HVTN

088, 205), University of Alabama at Birmingham IRB (HVTN 088, 205), Canton de Vaude Commission Cantonale d'ethique de la recherce (HVTN 096), Emory University IRB (HVTN 205), New York Blood Center IRB (HVTN 205), Fenway Community Health IRB (HVTN 205), Columbia University Medical Center IRB (HVTN 205), Partners Human Research Committee (HVTN 205), UCSF Committee on Human Research, (HVTN 205), Comite Institucional de Bio-Etica Asociacion Civil IMPACTA Salud y Educacion (HVTN 205).

Note that full information on the approval of the study protocol must also be provided in the manuscript.

Clinical data

Policy information about clinical studies

All manuscripts should comply with the ICMJE guidelines for publication of clinical research and a completed CONSORT checklist must be included with all submissions.

Clinical trial registration	HVTN 076: NCT00955006; HVTN 086: NCT01418235; HVTN 088: NCT01376726; HVTN 096: NCT01799954; HVTN 097: NCT02109354; and HVTN 205: NCT00820846
Study protocol	The full trial protocols may be access from the HIV Vaccine Trials Network upon request
Data collection	Clinical data and samples were collected according to the study protocol and at study sites as listed in clinicaltrials.gov. Binding Antibody data were collected at Duke University according to the study protocol and assay specific study plans.
Outcomes	Data reported in this manuscript were exploratory for each study protocol. The primary and secondary outcomes of each trial are defined in the study protocol and listed on clinicaltrials.gov
